# Across land, sea, and mountains: sulphate aerosol sources and transport dynamics over the northern Apennines†

**DOI:** 10.1039/d5ea00035a

**Published:** 2025-06-30

**Authors:** Manuel Bettineschi, Bruno Vitali, Arineh Cholakian, Dino Zardi, Federico Bianchi, Victoria Sinclair, Johannes Mikkola, Paolo Cristofanelli, Angela Marinoni, Martina Mazzini, Liine Heikkinen, Minna Aurela, Marco Paglione, Bertrand Bessagnet, Paolo Tuccella, Giancarlo Ciarelli

**Affiliations:** a Institute for Atmospheric and Earth System Research/Physics, Faculty of Science, University of Helsinki Helsinki 00014 Finland giancarlo.ciarelli@helsinki.fi; b Department of Civil, Environmental and Mechanical Engineering, University of Trento Trento Italy; c Laboratoire de Météorologie Dynamique (LMD), Ecole Polytechnique, IPSL Research University, Ecole Normale Supérieure, Université Paris-Saclay, Sorbonne Universités, CNRS UPMC Université Paris 06, Route de Saclay Palaiseau 91128 France; d Center Agriculture Food Environment - C3A, University of Trento Trento Italy; e National Research Council of Italy, Institute of Atmospheric Sciences and Climate (CNR-ISAC) Bologna 40129 Italy; f Department of Environmental Science, Bolin Centre for Climate Research, Stockholm University Stockholm Sweden; g Finnish Meteorological Institute Erik Palménin Aukio 1 Helsinki 00560 Finland; h Department of Physical and Chemical Sciences, University of L'Aquila L'Aquila Italy; i Center of Excellence in Telesensing of Environment and Model Prediction of Severe Events (CETEMPS), University of L'Aquila L'Aquila 67100 Italy

## Abstract

In this study, we combine aerosol observations with high-resolution Eulerian (WRF-CHIMERE) and Lagrangian (FLEXPART) modelling to investigate the source regions, emission sources, transport pathways, and chemical transformation of sulphate aerosols at the high-altitude Monte Cimone station during July 2017. Our analysis shows that marine air masses are linked to higher levels of sulphate at Monte Cimone. In particular, the sea plays a dominant role in enhancing the oxidation of sulphur dioxide (SO_2_) into sulphate due to prolonged exposure to elevated hydroxyl radical (OH) concentrations over the sea. At the same time, sensitivity simulations reveal that industrial emissions contribute significantly to sulphate levels at Monte Cimone, even when air masses have spent a long time travelling over the sea. Furthermore, examination of vertical atmospheric dynamics indicates that free tropospheric air masses favour higher concentrations of sulphuric acid likely due to lower condensation sink (CS) conditions in the free troposphere (FT). In contrast, boundary layer conditions were found to enhance the transport of dimethyl sulphide (DMS) oxidation products, meaning that, over the Mediterranean Sea, DMS and its oxidation products do not reach the FT efficiently. Our results highlight the complex interaction between marine and terrestrial sources, atmospheric chemistry, and transport mechanisms in shaping sulphate aerosol levels at high-altitude sites. They also provide valuable insights into sulphate sources and transport processes over large geographical areas.

## Abstract 2

Marine air masses enhance SO_2_ oxidation due to elevated OH concentrations over the sea, increasing sulphate levels at Monte Cimone. Industrial emissions remain the major source, even during sea-dominated transport.

## Graphical abstract

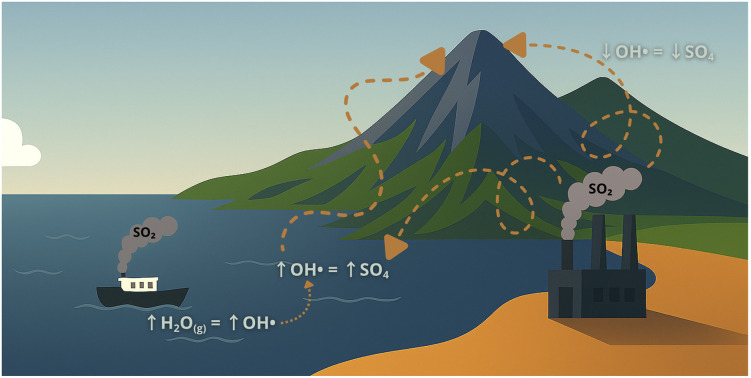

## 1 Introduction

Sulphate aerosols are among the main constituents of the total aerosol load in the atmosphere.^1–3^ They originate from a wide range of anthropogenic and biogenic sources and are largely produced in the atmosphere as a result of several chemical reactions, making them a not yet fully characterized secondary pollutant.^4,5^ Anthropogenic sources include mainly large-scale combustion processes, *e.g.* for energy production and manufacturing, as well as transportation systems, in particular ships. Biogenic emissions, on the other hand, include sea salt emissions, volcanic eruptions, and dimethyl sulphide (DMS) released from the ocean by phytoplankton.

Sulphate aerosols are largely formed through sulphur dioxide oxidation (SO_2_) by atmospheric oxidants such as hydroxyl radicals (OH) and by aqueous-phase oxidants such as hydrogen peroxide (H_2_O_2_), and ozone (O_3_) in cloud water and fog droplets. The product of SO_2_ oxidation by OH is the less volatile sulphuric acid (H_2_SO_4_) which is rapidly neutralized by atmospheric bases such as ammonia (NH_3_), to form the inorganic salt ammonium sulphate ((NH_4_)_2_SO_4_), which contains the majority of the sulphate ions (SO_4_^2−^) found in the atmosphere. In addition, SO_4_^2−^ ions in the atmosphere are also emitted as primary species, *via* the sea salt spray mechanism. Another important reaction pathway for SO_4_^2−^ includes the heterogeneous reaction of SO_2_ with transition metal ions (*i.e.*, Mn(ii) and Fe(iii)) which are often found in dust particles and can greatly increase the catalytic oxidation of S(iv) with oxygen dissolved in the aqueous phase. Such processes have been found to play an important role in fast sulphate growth during haze days in China.^6^

Oxidation of DMS can also contribute to aerosol particle formation and growth, and eventually influence the formation of cloud condensation nuclei (CCN). However, its oxidation mechanism in the atmosphere is still subject to substantial uncertainties.^7^ The estimated global DMS flux ranges from about 18 to 34 Tg S year,^8^ which accounts for half of the natural global atmospheric sulphur load.^9^ A previous study^10^ has shown that DMS and its oxidation products can be transported at high altitudes by Pacific Ocean air masses during the dry season after convective lifting over the remote Pacific Ocean to 6000–8000 m a.s.l., suggesting the potential impact of marine DMS emissions on the concentration of sulphur-containing vapours in the free troposphere, even at great distances from their emission sources, *i.e.* the ocean.

Oceans and sea regions are also heavily impacted by shipping traffic, with the Mediterranean Sea containing one of the busiest shipping routes in the world.^11^ Previous studies have revealed that in Europe the increase in PM_2.5_ concentration caused by shipping traffic is relatively limited.^12,13^ However, in the Mediterranean region the impact of ship emissions on PM_2.5_ concentration is more significant, with 5 to 20% of the total PM_2.5_ concentration related to this source.^14,15^ A recent multi model evaluation study, used five chemical transport models, namely CHIMERE, EMEP – European Monitoring and Evaluation Programme model, LOTOS-EURO, CAMx – Comprehensive Air Quality Model with Extensions and CMAQ – Community Multiscale Air Quality model, to study the potential impact of shipping on particle species in the Mediterranean Sea. The study revealed that SO_4_^2−^ was the main contributor to both the absolute ship-related PM_2.5_ and total PM_2.5_ concentrations with ship-related SO_4_^2−^ concentrations making up 44.6% of the PM_2.5_ concentration in the model ensemble mean. Additionally, emissions from the marine transport sector are also one of the least-regulated anthropogenic emission sources despite the recent introduction of strict limits on the maximum sulphur content in marine fuels in SECAs (sulphur emission control areas) and in EU ports. In the Mediterranean Sea, the Marine Environment Protection Committee (MEPC) introduced a sulphur emission control region on 1 January 2025, where the limit for sulphur in fuel oils used on board ships is 0.10%.^16^

High-altitude measurement sites are widely used to study the free troposphere (FT), providing a unique opportunity for long-term *in situ* observations with high temporal resolution.^17^ Their strategic locations are intended to minimize the influence of anthropogenic emission from the planetary boundary layer (PBL), allowing for more representative measurements of aerosols and trace gases in pristine environments. These observations are essential for understanding long-range transport mechanisms, atmospheric chemistry, and climate-related processes. Examples of such stations are the Jungfraujoch station in the Swiss Alps, the Chacaltaya station in the Bolivian Andes, the Izaña station in Tenerife, and the Nepal Climate Observatory-Pyramid station in the Himalayas, among others. These high-altitude sites have been the focus of many observational studies (*e.g.* ref. 18–22), providing valuable *in situ* measurements of atmospheric composition and dynamics. They have also played a key role in supporting modelling studies that improve our understanding of atmospheric processes on both regional and global scales (*e.g.* ref. 23–27).

The CMN GAW/WMO Global Station, situated at 44°11′N, 10°42′E, and at an elevation of 2165 meters above sea level, is located on top of Monte Cimone, the highest peak of the Northern Apennines in Italy. This location marks the transition between the continental European climate to the north and the Mediterranean climate to the south. The main ridge of the mountain runs in a NW–SE direction, nearly parallel to the Tyrrhenian coast, which lies approximately 50 km to the south-west. The surrounding valleys are oriented perpendicularly to the ridge on the northeastern side and both perpendicularly and parallel on the southwestern side. The station is positioned on the summit of the mountain, offering a 360°-wide unobstructed horizon. Due to its high elevation and significant distance from major pollution sources, the CMN station serves as an ideal platform for studying the chemical and physical characteristics, as well as the climatology, of the FT over southern Europe and the Northern-Central Mediterranean Basin. In summer, during daytime, the site is typically considered to be influenced by air masses from the PBL, while at night, it is considered to predominantly represent the FT.^28^

Our previous modelling study^26^ over the area showed that sulphate and organic aerosols contribute to about 80% of the total aerosol load during the investigated time period, *i.e.* July 2017, with biogenic aerosols being the main component of the total organic aerosol fraction. The results also confirmed that the model adopted was suitable for capturing effects typically occurring in high-complex topography environments, such as the thermally-driven flows, typical of sea and mountain regions, in line with available observational data. The study focused mainly on near-surface meteorological dynamics over complex terrain and how these affect the transport of organic aerosols. However, it did not investigate the sources and transportation processes of sulphate aerosols.

In this study, we build on our previous high-resolution application of the WRF-CHIMERE v2020r3 model over the northern Apennines in July 2017, now combined with the WRF-FLEXPART v3.3.2 model. The objectives of this study are to (i) identify the areas (source-regions) most affecting sulphate aerosol concentration at the high-altitude research station of CMN, (ii) perform source apportionment of sulphate aerosols at CMN to quantify the contribution of different emission sources (*i.e.* industries, ships, DMS, and sea salt), (iii) study the role of the Mediterranean Sea on sulphate production, and transport at CMN, and (iv) study the influence of free-tropospheric air masses on sulphur-containing species.

The manuscript is organized as follows. Section 2 describes the methods, the WRF-CHIMERE v2020r3 and WRF-FLEXPART v3.3.2 models and their setups for the study. In Section 3 the results and discussion of the air mass history analysis, the source apportionment of sulphate aerosols, the analysis of the marine transportation of SO_4_^2−^, as well as an analysis of the effect of the FT on concentrations at CMN are presented. In Section 4 we report the main conclusions of the study.

## 2 Data and methods

To study the sources and air mass history of sulphate aerosols over the northern Apennines we combined high resolution aerosol observational data with simulations performed with an Eulerian (WRF-CHIMERE) and a Lagrangian model (FLEXPART). Specifically, we use the SO_4_^2−^ concentration measured using an Aerosol Chemical Speciation Monitor (ACSM)^29^ at the Ottavio Vittori Observatory. The ACSM measured the non-refractory sub-micrometer particulate matter mass with an aerodynamic diameter less than 1 μm (PM_1_), and provided the concentrations of organics, nitrate, sulphate, ammonium and chloride. The practical time resolution was 30 minutes. A detailed explanation of the WRF-CHIMERE simulations used in this study is provided in ref. 26. Here, we will briefly describe the aspects of the WRF-CHIMERE setup relevant to this study in Section 2.1. Section 2.2 introduces the FLEXPART model and the simulation setup. Sections 2.3, 2.4, 2.5, and 2.6 report the analysis approaches, which integrate observational data with both the WRF-CHIMERE and FLEXPART models.

### 2.1 The WRF-CHIMERE model

The WRF-CHIMERE v2020r3 model^30^ is a three-dimensional online chemical transport model, coupled *via* the OASIS3 external coupler,^31^ which allows to account for the physical and chemical processes occurring in the Earth's atmosphere, from the emission of both anthropogenic and biogenic pollutants at different elevations, to chained chemical reactions of a multitude of chemical compounds, to dry and wet deposition processes. Along with several other chemical transport models, it serves as a member of the Copernicus Atmosphere Monitoring Service's (CAMS) for European air quality forecasts (https://atmosphere.copernicus.eu/).

This study uses the same simulations as those performed by the authors of ref. 26, who performed a 1-month long high-resolution simulation covering all of July 2017. The simulation had four nested domains centered over the CMN station. The largest domain had a resolution of 27 × 27 km while the smallest had a resolution of 1 × 1 km (see Fig. A1 in the ESI†). Meteorological fields were calculated using the WRF regional model (v3.71),^32^ forced with Climate Forecast System (CFSv2) data from the National Centers for Environmental Prediction. Simulations were performed using the Rapid Radiative Transfer Model radiation scheme,^33^ the Thompson aerosol-aware microphysics scheme to treat the microphysics,^34^ the Monin–Obukhov surface-layer scheme,^35^ and the NOAA Land Surface Model scheme for land surface physics.^36^ The boundary-layer option was the Mellor–Yamada–Janjic turbulent kinetic energy (TKE) scheme.^37^ WRF simulations were performed on 33 vertical sigma layers.

#### 2.1.1 Anthropogenic emissions

Anthropogenic emission fluxes of SO_2_, nitrogen oxides (NO_*x*_), black carbon (BC), organic carbon (OC), carbon monoxide (CO), ammonia (NH_3_) and non-methane volatile organic compounds (NMVOCs) were retrieved from the CAMS datasets for the year 2017 at 0.1 × 0.1 degree (around 10 km) horizontal resolution and at hourly time resolution over the investigated period (July 2017). These emission fluxes were then down-scaled to the high resolution grid (1 km) using proxy data in a top–down approach (a mass-conservative algorithm funnelling industrial and traffic emissions into grid cells containing industrial sources or the road-map). Anthropogenic emissions were divided into sectors based on the GNFR (Grouped Nomenclature for Reporting) classification. Details about the individual sectors are reported in Table S1 in the ESI†.

#### 2.1.2 Sea salt emissions

In CHIMERE, sea salt emission parameterizations compute the flux of sea salt particle numbers 
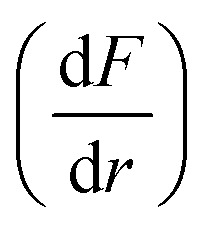
 as a function of particle radius (*r*) and 10-meter wind speed (U_10_). Different sea salt emission schemes are implemented in CHIMERE, for this study we selected the parametrization presented in ref. 38 combined with the parameterization from ref. 39 for the size distribution (*B*).1
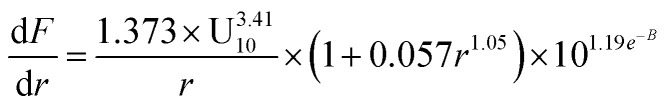

2 *B* = *d*_4_·*r*^4^ + *d*_3_·*r* + *d*_2_·*r* + *d*_1_·*r* + *d*_0_

where *d*_*i*_ represents coefficients.

Sea salts particles influence SO_4_^2−^ concentrations through multiple pathways. Firstly, sea salts particles contain a small fraction of SO_4_^2−^. Additionally, sea salts particles are highly hygroscopic leading to substantial water uptake, thus enhancing the aqueous-phase dissolution of SO_2_ and its oxidation by soluble oxidants. Finally, sea salt particles provide additional surface area, affecting the gas–particle partitioning of inorganic gases like H_2_SO_4_.

#### 2.1.3 DMS emissions

In CHIMERE, the DMS (dimethyl sulphide) emission parametrization computes the emission flux as a function of U_10_ and the DMS sea surface concentration. Here we selected the scheme described in ref. 40 where DMS emission fluxes (*F*_DMS_) are affected by the water concentration of DMS (*C*_w_) at the surface of the sea, U_10_ and the temperature (*T*):

3 *F*_DMS_ = *K*_w_(T,U_10_) × *C*_w_

where *K*_w_ is the transfer velocity which depends on *T* and U_10_, and it is calculated as follows:4
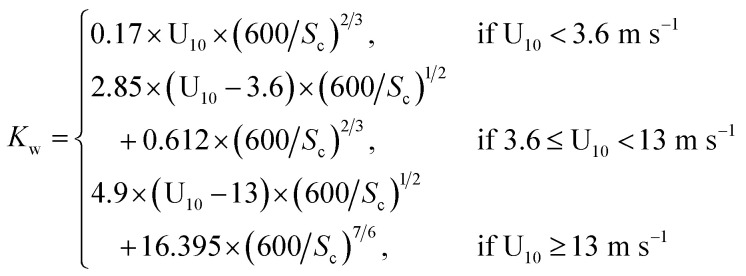
where *S*_c_ is the Schmidt number, which depends on *T*.

The *C*_w_ input data were retrieved from ref. 8 and regridded on the simulation domain directly by CHIMERE.

#### 2.1.4 Sulphur chemistry

The chemical mechanism used for the gas-phase chemistry is the MELCHIOR2 scheme,^41^ which includes the gas phase oxidation of SO_2_ by OH. For the inorganic aerosol constituents, the ISORROPIA thermodynamic model was used to calculate the partitioning between gas and particle phases.^42^

The aerosol option of the model was selected, thus incorporating aqueous sulphate chemistry. In CHIMERE sulphate is produced through the following net aqueous reactions, as described in ref. 43–45:

5 SO_2(aq)_ + O_3(aq)_ → SO_4(aq)_^2−^

6 HSO_3(aq)_^−^ + O_3(aq)_ → SO_4(aq)_^2−^

7 SO_3(aq)_^2−^ + O_3(aq)_ → SO_4(aq)_^2−^

8 SO_2(aq)_ + H_2_O_2(aq)_ → SO_4(aq)_^2−^

9 SO_2(aq)_ + NO_2(aq)_ → SO_4(aq)_^2−^

where SO_2_, H_2_O_2_, and O_3_ in the aqueous-phase are in equilibrium with the gas-phase concentrations. Moreover, aqueous SO_2_ is dissociated into HSO_3_^−^ and SO_3_^2−^. Additionally, catalysed oxidation reactions of sulphur dioxide in aqueous droplets, involving iron and manganese, are included in CHIMERE, as described in ref. 44. However, no dust speciation was included in these simulations, meaning that practically the catalysed oxidation reactions were not active. Henry's law coefficient and other aqueous equilibrium constants are applied as outlined in ref. 2.

A simplified chemistry of DMS is also available in CHIMERE; the DMS chemical scheme is the one presented in ref. 46, with only gas-phase reactions currently implemented and including the reactions reported in Table 1.

**Table 1:** List of chemical reactions involving DMS oxidation pathways and their corresponding kinetic rate expressions used in this study

Reactions	Kinetic rates (molecules cm^−3^ s^−1^)
DMS + OH → 0.997 (SO_2_ + CH_3_O_2_ + HCHO) + 0.003 (MSA + HCHO)	*k*(*T*) = *A* exp(−*B*/*T*), *A* = 1.13 × 10^−11^, *B* = 253
DMS + OH → DMSO	*k*(*t*) = *A*_1_ exp(−*B*_1_/*T*) × *M*/(1 + *A*_2_ exp(−*B*_2_/*t*) × *M*), *A*_1_ = 1.7 × 10^−42^, *B*_1_ = 7810, *A*_2_ = 5.5 × 10^−31^, *B*_2_ = 7460, *M* = (80% N_2_ + 20% O_2_)
DMS + NO_3_ → SO_2_ + HNO_3_ + CH_3_O_2_ + HCHO	*k*(*T*) = *A* exp(−*B*/*T*), *A* = 1.9 × 10^−13^, *B* = −500
DMSO + OH → MSIA + CH_3_O_2_	*k* = 9 × 10^−11^
MSIA + OH → CH_3_O_2_ + SO_2_ + H_2_O	*k* = 9 × 10^−11^

### 2.2 The FLEXPART model

The FLEXible PARTicle dispersion model (FLEXPART) is a Lagrangian model used to simulate both the forward and backward dispersion of particles. In this study, we used FLEXPART version 3.3.2 (ref. 47) in backward mode to trace the origins of air masses arriving at the CMN site. Simulations were driven by meteorological data generated from the WRF simulations (the same used for the CHIMERE simulations), with a temporal resolution of 15 minutes.

The FLEXPART model was configured with one domain matching the resolution and region covered by the outermost domain of the WRF-CHIMERE simulations, with a resolution of 27 × 27 km^2^. This domain was designed to capture long-range transport processes. The simulation was setup with 12 vertical levels extending from the surface to 9000 meters above ground level. The layer thickness follows a pseudo-exponential distribution, with finer resolution (50 meter) near the surface and progressively increasing layer depth with altitude.

Between July 4 and July 28, 2017, we released 10 000 particles per hour from CMN and tracked their back trajectories over 72 hours. The passive tracer particles were emitted from altitudes ranging from 0 to 100 meters above ground level. The output of FLEXPART in backward mode is the Source–Receptor Relationship (SRR), expressed in units of seconds, which can be interpreted as a proxy of the time the particles spent in each grid cell.

### 2.3 SO_4_^2−^ source apportionment

In order to determine the relative contribution of the different sources to the SO_4_^2−^ concentrations at CMN, we performed five WRF-CHIMERE sensitivity simulations. We zero-tagged, over all four domains, the emissions of the following sectors/sources: industrial combustion, ship traffic, DMS, and sea salt. For industrial combustion, as well as ship traffic, we specifically zero-tagged emissions of SO_2_, and SO_4_^2−^. Additionally, we also separated the contribution of the boundary conditions (*i.e. trans*-boundary transport) by zeroing the SO_2_ “injected” at the boundary into the first domain. In our case, boundary conditions for aerosols and gas-phase constituents were retrieved from the climatological simulations of LMDz-INCA3 (ref. 48) and the GOCART model.^49^

### 2.4 Source region contribution

To investigate the impact of air mass history on SO_4_^2−^ concentrations at CMN, we combined SO_4_^2−^ observations from the CMN station with FLEXPART output. We calculated the Source Region Contribution (SRC), which assigns to each grid cell the average SO_4_^2−^ concentration at CMN across all times when air masses that passed over that grid cell during the previous 72 hours arrived at the station. In other words, each time an air mass reaches CMN, if it previously intercepted a given grid cell, the sulphate concentration measured at CMN is “assigned” to that cell. Averaging these values over all such arrival events gives the SRC, revealing how strongly each grid cell is linked to the sulphate levels observed at CMN. This method provides valuable insights into how air masses from various geographical regions contribute to sulphate concentrations at CMN, helping to identify key source-regions responsible for elevated SO_4_^2−^ levels. More specifically, given a simulation domain Ω, containing simulation time (*t*), height (*h*), longitude (*x*) and latitude (*y*) as coordinates, and an air mass arrival time (*τ*), the SRC was calculated assigning the SO_4_^2−^ concentration recorded during *τ*, to all the (*x*, *y*) grid points intercepted by at least one particle (at any height) in the 72 hours prior to the release. A summation was then performed over all the releases; then, for each (*x*, *y*) pair this value was divided by the number of trajectories that intercepted the given (*x*, *y*) grid point. This can be mathematically described using the following equations:10
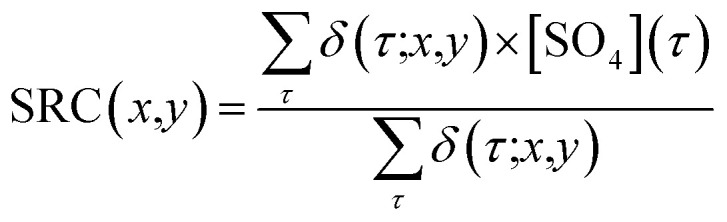
11
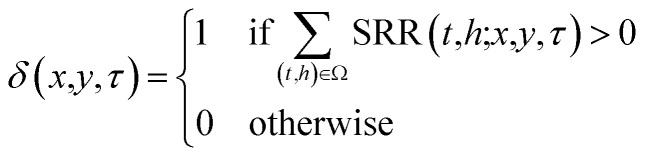


### 2.5 Air mass exposure and time over sea

To investigate the effects of various properties of different air masses, we adapted the concept of Air Mass Exposure (AME), as introduced in ref. 50. AME calculations combined FLEXPART output with two-dimensional fields (*e.g.*, emissions fields or population density) to determine when air masses were exposed to emissions from different pollutants. Here, we modified the AME calculations by integrating the FLEXPART output with the four-dimensional output from the CHIMERE model. For each release event, we calculated the AME to OH (AME_OH_), with the goal of obtaining insights into the potential for pollutants transported within the air masses to undergo oxidation. The OH concentration used in these calculations was derived from the WRF-CHIMERE model output. This modification allows us to capture the temporal and vertical variability in OH concentrations within the air masses, offering a more detailed analysis of the potential for SO_4_^2−^ formation through OH oxidation. The AME_OH_ for a specific *τ* is calculated according the following equation:12
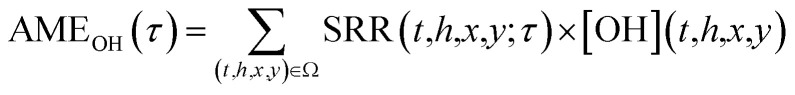


Additionally, to determine whether air masses with greater exposure to the marine boundary layer (MBL) affect SO_4_^2−^ concentrations at CMN, we calculated the time each air mass spent over the sea at altitudes below 500 meters. This metric allowed us to assess the extent to which exposure to MBL contributes to SO_4_^2−^ levels at the measurement site, providing further insights into the role of sea-sourced emissions and marine air interactions. The 500 m threshold was chosen to ensure that the air masses were exposed to the MBL while avoiding as much as possible influence from the FT. In Fig. A2 in the ESI† we can see the average MBL height for the simulation period used to choose the 500 m threshold. By choosing a pseudo-MBL with a constant depth of 500 m, we neglected the variation in MBL height. Since the diurnal variation in the MBL is not as pronounced as in the PBL over land,^51^ we considered this a reasonable approximation for our analysis.

The time over sea below 500 meters (ToS_500_) for a specific *τ* is calculated as follow:13
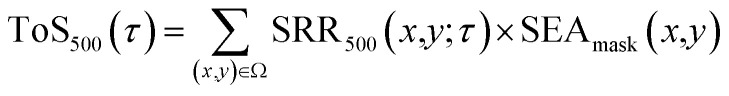
14
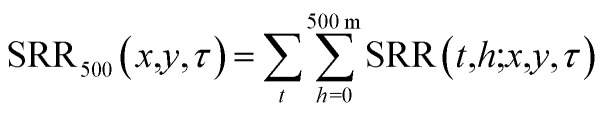
where SEA_mask_ is equal to 1 over sea and 0 over land.

To evaluate the MBL influence on SO_4_^2−^, we classified air masses based on ToS_500_ into three categories (low, medium, and high). Our method first divided the dataset into 10 deciles based on the ToS_500_ data, each containing an equal number of data points, and we plotted the distribution of SO_4_^2−^ for each decile (see ESI Fig. A3†). By visually inspecting these decile distributions, we identified natural groupings and consolidated them into three broader categories: low (0–30th percentile), medium (30–90th percentile), and high (90–100th percentile). These categories roughly correspond to ToS_500_ values of <3 hours (low), 3–14 hours (medium), and >14 hours (high). To statistically validate these groupings, we performed a Kruskal–Wallis test,^52^ followed by a Dunn–Bonferroni post-hoc test,^53^ confirming that the three categories are statistically significantly different (more details in the results section). This approach, although initially subjective, effectively captures the distinct SO_4_^2−^ distribution patterns observed in the data.

### 2.6 Free troposphere influence

In order to understand what is the influence of the FT on CMN, we calculated the FT influence (FT_SRR_) for every air mass arriving at CMN, as in ref. 25.15
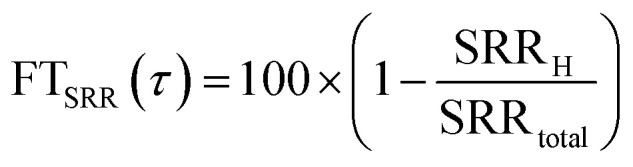
16
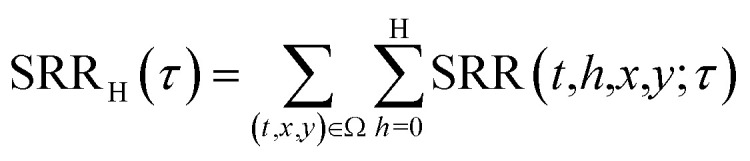
where SRR_total_ is equal to the theoretical total residence time of the simulation expressed in seconds (72 *h* = 259 200 s). This method assumes a pseudo-boundary layer with a constant depth H, neglecting the diurnal variation in the PBL height. In this study, we additionally calculated the FT_SRR_ using the PBL height values from WRF as the threshold H in eqn 16). Specifically, for a given *τ* we use a different threshold H for every of the previous 72 hours, calculated as follows:17
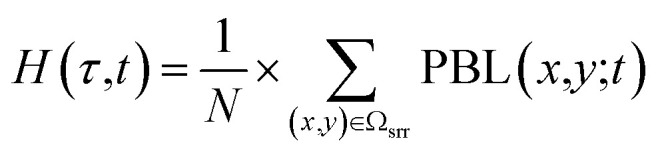
where Ω_srr_ contains all the grid points where the SRR for the release *τ* is not zero at time *t*, and *N* is the number of these grid points. In other words, for every hour prior to the release we calculated the average PBL height between the points with a positive SRR (*i.e.* where the air mass is located at that moment).

We classified air masses based on FT_SRR_ into three categories: low, medium, and high. The high category corresponds to FT_SRR_ values greater than 92%, the low category includes values below 75%, and the medium category falls in between. These thresholds were determined based on Fig. A4 in the ESI.† The “high” category is clearly distinguishable due to the presence of a secondary peak in the probability density function of FT_SRR_, and these air masses can be interpreted as representative of almost pure FT conditions. The “low” category threshold was chosen to lie between the main peak of the probability density function and the lowest observed value. This category represents air masses more exposed to the PBL compared to the typical air mass condition (“medium” category). However, since FT_SRR_ remains above 50% in basically all cases, every air mass arriving at CMN is at least 50% exposed to the FT, meaning that the “low” category is still predominantly influenced by the FT.

## 3 Results and discussion

An in-depth evaluation of the WRF-CHIMERE simulations used in this study was presented in ref. 26. The evaluation of the modelled SO_4_^2−^ and SO_2_ are reported in Fig. A5 of the ESI,† indicating a good agreement between the model and the observations. Here, in Section 3.1, we present the results of the FLEXPART output analysis. Sulphate source apportionment results are shown in Section 3.2. The influence of marine transport is presented in Section 3.3. Finally, a comparison with the high altitude research station of Chacaltaya, is reported in Section 3.4.

### 3.1 Analysis of air mass history

In order to understand the origin and transport pathways of SO_4_^2−^ measured at the CMN site, an analysis of air mass history was conducted. This section presents the results of the air mass history analysis, which includes spatial distribution of all the particles released and the influence of different air mass source regions on SO_4_^2−^ concentrations at the site.

**Fig. 1:**
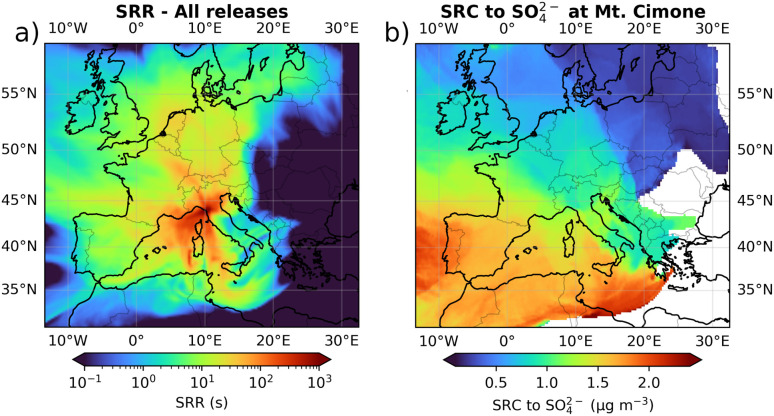
(a) SRR sum for all the releases during the 04-28 July 2017 period. (b) Source region of the SO_4_^2−^ observed at CMN. The SRC was calculated by assigning the SO_4_^2−^ concentration recorded during the release, to all the grid cells intercepted by at least one particle in the 72 hours prior to the release.

Fig. 1(a) shows the SRR summed over all vertical layers and over all the particle releases conducted between 04 and 28 July 2017. The SRR represents the influence of different regions on the air mass arriving at CMN, as calculated using backward trajectories. The highest SRR values are concentrated over two key areas: the Mediterranean Sea and the Po Valley basin. This suggests that these regions were the dominant source-areas influencing CMN during the analysed period. Specifically, approximately 60% of the time, air masses originated from or passed over the Mediterranean Sea, while 40% arrived from the Po Valley side. The SRR distribution suggests the potential for a significant marine influence on the air reaching CMN, bringing aerosols that originated from or were exposed to the marine environment, while the influence from the Po Valley region is more likely associated with anthropogenic emissions from industrial activities.

The source region of SO_4_^2−^, calculated through the SRC is shown in Fig. 1(b). The results indicate that the south-western Mediterranean is the primary source region for SO_4_^2−^ at CMN, suggesting that sea exposed air masses could be the predominant contributors to the observed SO_4_^2−^ concentrations. Surprisingly, despite significant anthropogenic emissions from industrial activities in the Po Valley, this region does not appear to contribute substantially to elevated SO_4_^2−^ levels. This may imply either low SO_2_ emissions in the Po Valley, limited transformation of SO_2_ from the Po Valley into sulphate during transport, or that other processes, such as dry deposition, diminish its impact by the time the air masses reach CMN.

**Fig. 2:**
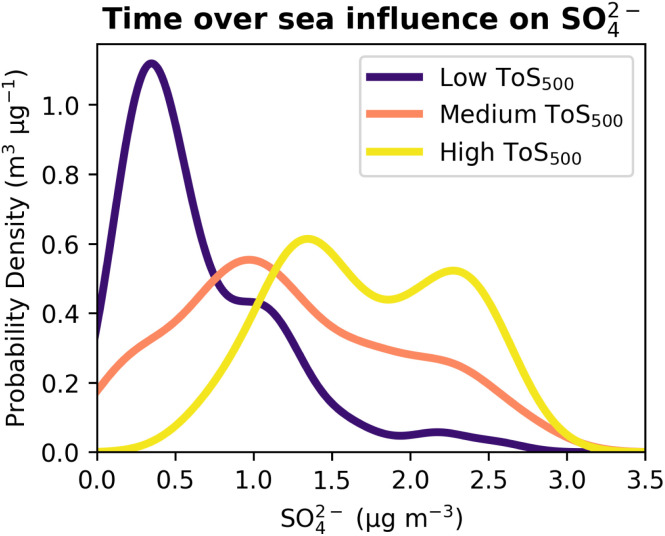
SO_4_^2−^ distribution at CMN based on the number of hours air masses spent above the sea (below 500 meters altitude) during the 72 hours prior to reaching the site.

To further evaluate the marine influence on SO_4_^2−^, we classified air masses based on ToS_500_ into three categories as described in the methods section: low, medium, and high ToS_500_. Fig. 2 shows the SO_4_^2−^ concentration distributions for these three categories. It is immediately apparent that air masses with low ToS_500_ are associated with lower SO_4_^2−^ concentrations at CMN, while those with high ToS_500_ correspond to higher concentrations. To determine whether these differences were statistically significant, we applied a Kruskal–Wallis test. This non-parametric test was chosen because it does not assume a normal distribution of SO_4_^2−^ concentrations and is appropriate for comparing multiple independent groups. Since the Kruskal–Wallis test indicated significant differences between the groups (*p*-value = 2 × 10^−27^), we followed it with a Dunn-Bonferroni *post hoc* test to identify which pairs of distributions were different. The results show that all pairwise comparisons between the distributions were statistically significant, with *p*-values <10^−5^ for each pair.

These results strongly indicate that the marine influence on SO_4_^2−^ concentrations at CMN is significant, with higher concentrations associated with longer time spent over the sea. Furthermore, these results are in line with the synoptic context described in ref. 26 where synoptic phases with strong westerly winds over the Mediterranean and CMN, roughly correspond to periods with higher SO_4_^2−^ concentrations.

### 3.2 Zero-out source apportionment

To gain a deeper understanding of the sources contributing to the SO_4_^2−^ levels, we use model-based approaches (zero-out simulations) to quantify the contributions of various sectors, including industries, ships, sea salt, DMS, and long range transport (boundary conditions) to SO_4_^2−^ concentrations at CMN. This is achieved by analysing the impact of each sector's emissions on SO_4_^2−^ levels through a series of sensitivity simulations.

**Fig. 3:**
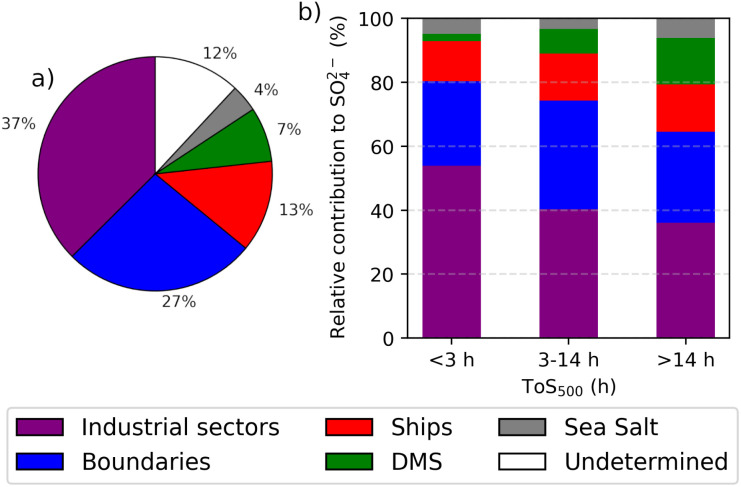
(a) Model-based SO_4_^2−^ source apportionment at CMN. The contribution of each sector was estimated by removing SO_4_^2−^ precursor emissions for each individual sectors. The resulting mean SO_4_^2−^ concentration from each modified simulation was then subtracted from the mean SO_4_^2−^ concentration of the base simulation to determine the sectoral contribution. (b) Model-based SO_4_^2−^ source apportionment at CMN for each ToS_500_ class. In this case the undetermined category is not shown.

**Fig. 4:**
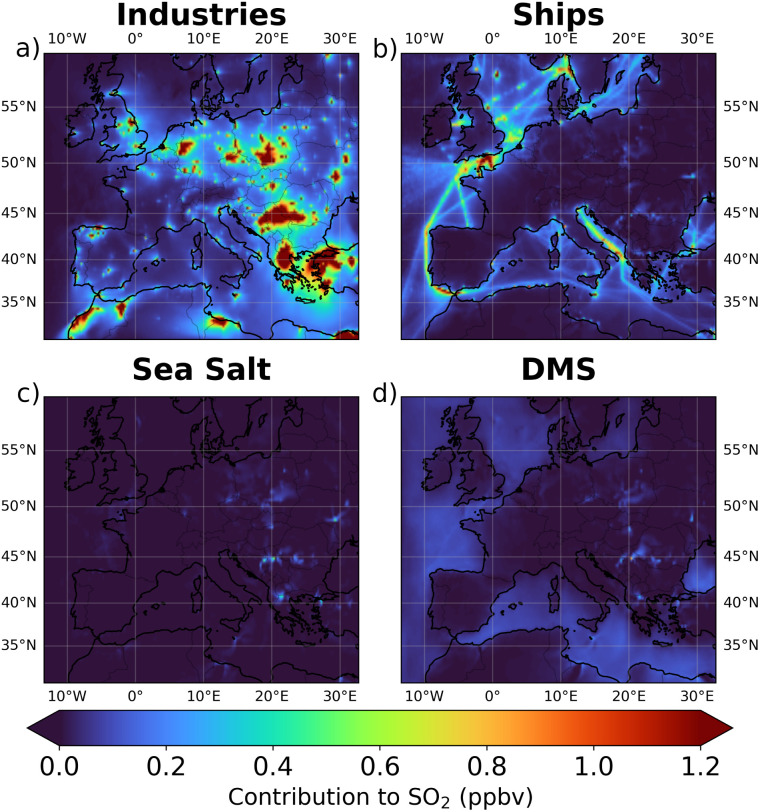
Average contribution of industries (a), ships (b), sea salt (c), and DMS (d) to SO_2_ concentration. Sectoral contributions were calculated by subtracting the mean SO_2_ concentration from each modified simulation from the mean SO_2_ concentration of the base simulation. The mean concentrations were calculated within the first six layers of the model, corresponding to altitudes between 0 and 500 meters above ground level.

Fig. 3(a) presents the results of the model-based SO_4_^2−^ source apportionment at CMN. On average, the industrial sector is the largest contributor to SO_4_^2−^ concentrations at CMN, accounting for 37% of the total. This is followed by contributions from the boundaries, which represent 27% of the total. Marine sources collectively contribute 25%, with ships contributing 13%, DMS 7%, and sea salt 4%. The remaining 12% could not be attributed to any specific sector, due to non-linear interactions in the model.^54^ These non-linearities are likely caused by aerosol feedback on meteorology. Fig. A6 in the ESI† shows the average variation in the wind components between the baseline simulation and all sensitivity simulations. The most significant variations are present in the “industries off” and “sea salt off” simulations. Overall, the root mean square difference in wind components across all data points of the simulations (*x* × *y* × *h* × *t*) ranges from a maximum of 0.84 to a minimum of 0.53 m s^−1^. While we cannot ignore the impact of these non-linearities on the results, Fig. 4c shows that their overall effect on actual concentrations is small. Since sea salt should not influence SO_2_, we can use this as a reference to quantify the effects of these non-linearities. The results indicate that these effects are generally small (compared to the emission effect) and localized, and they should not impact the overall conclusions of this study.

**Fig. 5:**
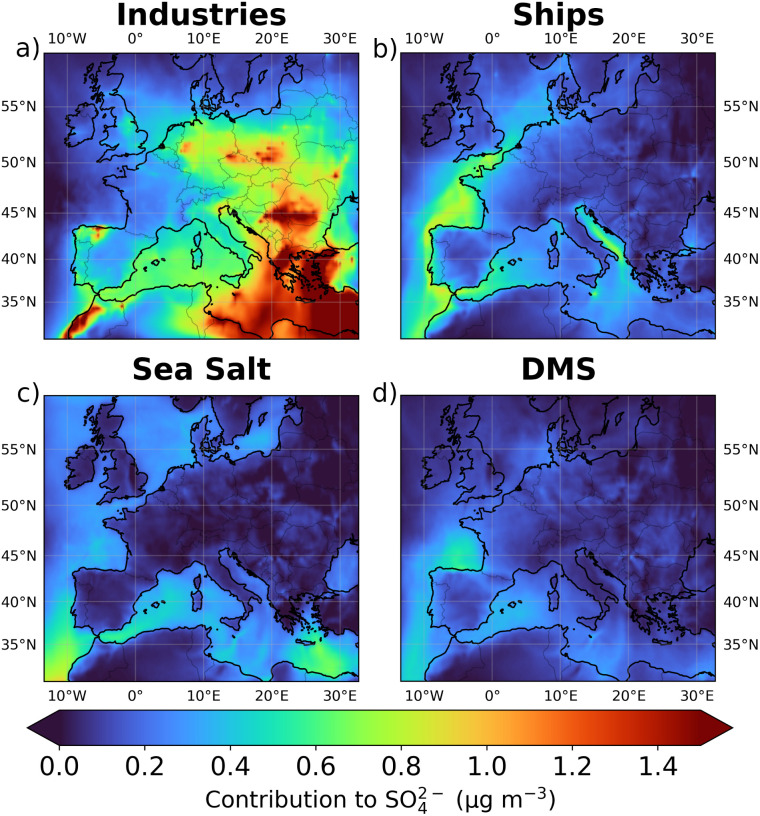
Average contribution of industries (a), ships (b), sea salt (c), and DMS (d) to SO_4_^2−^ concentration. Sectoral contributions were calculated by subtracting the mean SO_4_^2−^ concentration from each modified simulation from the mean SO_4_^2−^ concentration of the base simulation. The mean concentrations were calculated within the first six layers of the model, corresponding to altitudes between 0 and 500 meters above ground level.

The high contribution of industries to SO_4_^2−^ concentrations may seem surprising given the results presented in the previous section (*i.e.* the ToS_500_ influence over SO_4_^2−^ concentration and the frequency of air masses from the sea). However, Fig. 4 and 5 offer a possible explanation. These figures show the average contributions of industries (a), ships (b), sea salt (c), and DMS (d) to SO_2_ and SO_4_^2−^ concentrations across the study domain. It becomes clear that industrial emissions can significantly influence SO_2_ and SO_4_^2−^ levels even over the sea, despite the absence of direct industrial emissions in these areas. Specifically, over the sea, between sea level and 500 meters above sea level, the average contribution to SO_2_ is 0.15 ppbv from industries (dominated by the south Mediterranean region), 0.13 ppbv from ships, 0.05 ppbv from DMS, and as expected 0 ppbv from sea salt. Similarly, the average contribution to SO_4_^2−^ over the sea is 0.65 μg m^−3^ from industries, 0.25 μg m^−3^ from ships, 0.16 μg m^−3^ from DMS, and 0.29 μg m^−3^ from sea salt. This is also confirmed by Fig. 3(b), which illustrates the source apportionment (without the undetermined component) for each of the three ToS_500_ classes, and it shows that the relative contribution of SO_4_^2−^ originating from industries is significant (roughly 1/3 of the total) even when the air masses spend a long time over the sea.

### 3.3 Dynamics of sulphate aerosols over sea areas

In the previous sections, we showed that industrial emissions can significantly contribute to SO_4_^2−^ concentrations recorded at CMN, despite the site being primarily influenced by air masses originating from the sea sector. In this section, we aim to explore this topic in more detail, focusing on how industrial sulphate aerosols are transported across the sea before reaching the Northern Apennines.

**Fig. 6:**
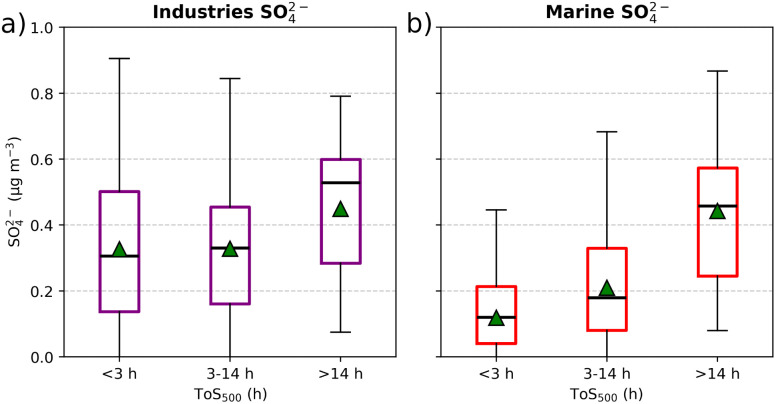
Boxplots showing the distribution of sectoral contributions to SO_4_^2−^ concentrations based on the number of hours air masses spent above the sea (below 500 meters altitude) during the 72 hours prior to reaching CMN. Panel (a) represents SO_4_^2−^ originating from industries emissions, while panel (b) shows SO_4_^2−^ originating from marine emissions (ships, sea salt, and DMS). The central line in each box represents the median, the green triangle represents the mean, the box edges represent the interquartile range (IQR), and the whiskers extend to the smallest and largest values within 1.5 times the IQR. Any points outside the whiskers are considered outliers.

Fig. 6 shows the SO_4_^2−^ at CMN originating from industries (a) and from marine (DMS + sea salt + ships) sources (b) categorized by the low, medium, and high ToS_500_ classes introduced in Section 3.1. The influence of marine sources increases with higher ToS_500_, as air masses are more exposed to emissions from ships, DMS and sea salt. In contrast, the contribution from industrial sources shows less variation across the three classes. SO_4_^2−^ levels remain relatively consistent between the low and medium ToS_500_ classes, while the high ToS_500_ class shows an increase in SO_4_^2−^ levels.

**Fig. 7:**
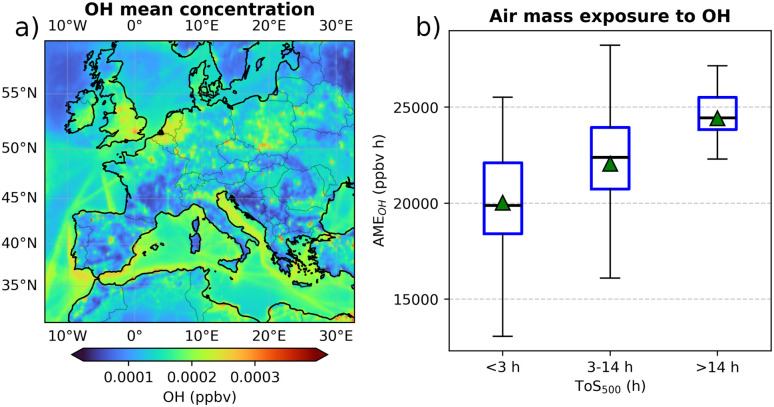
(a) OH mean concentration during the simulation period. The mean concentration was calculated within the first six layers of the model, corresponding to altitudes between 0 and 500 meters above the ground level. (b) Air mass exposure to OH based on the number of hours air masses spent above the sea (below 500 meters altitude) during the 72 hours prior to reaching CMN. The central line in each box represents the median, the green triangle represents the mean, the box edges represent the IQR, and the whiskers extend to the smallest and largest values within 1.5 times the IQR. Any points outside the whiskers are considered outliers.

Since no industrial emissions are located over the sea, this increase could be caused by more favourable conditions for SO_4_^2−^ production through SO_2_ oxidation in marine environments. Fig. 7 indicates that, on average, OH concentrations are higher over the sea than over land (a). Additionally, panel (b) presents the AME_OH_ for the three ToS_500_ classes. These boxplots clearly demonstrate that air masses spending more time over the sea tend to have higher exposure to OH, which should result in greater SO_2_ oxidation.

**Fig. 8:**
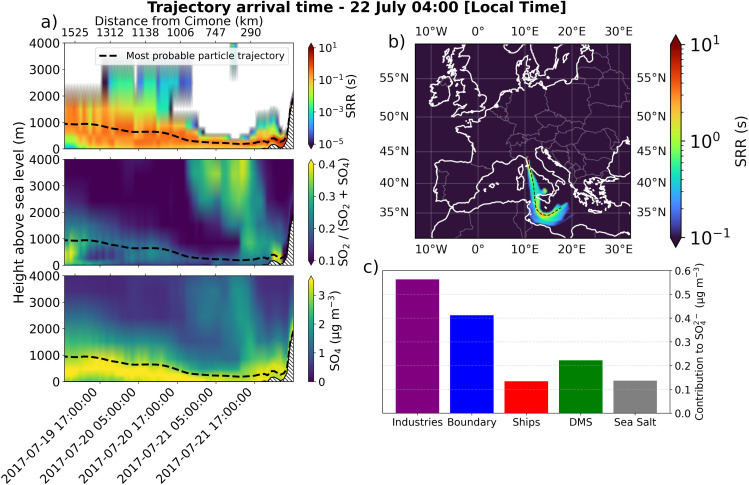
An example of an air mass arriving from the sea is shown. (a) The vertical profile along the most probable trajectory, with SRR in the (a) top panel, S_ratio_ in the (a) middle panel, and SO_4_^2−^ in the (a) bottom panel. The 2D SRR in (b) highlights the origin of the air mass, the black dashed line represents the most probable trajectory. (c) The absolute contribution of individual sources to SO_4_^2−^ for this specific case.

A specific example of an air mass arriving from the marine sector is presented in Fig. 8. Panel (a) illustrates the SRR, SO_2_/(SO_2_ + SO_4_^2−^) ratio (S_ratio_), and the SO_4_^2−^ concentration along the most probable trajectory calculated by FLEXPART for the air mass arriving July 22 at 4:00 AM local time. Panel (b) highlights the area of origin of the air mass through the SRR summed over the vertical dimension. Panel (c) shows the contribution of different sources to SO_4_^2−^ at CMN at the time of the arrival of the air mass. The figure reveals that along the air mass trajectory the S_ratio_ tend to decrease when travelling over the sea, suggesting that SO_2_ is being converted in SO_4_^2−^. This trajectory is not necessarily the most representative of the entire period but was chosen because it remains in contact with the MBL for nearly the entire 72-hour period. As a result, it is expected to be significantly influenced by marine sources of SO_4_^2−^. However, despite this prolonged exposure, industrial contributions remain the dominant contributor to SO_4_^2−^ at CMN.

These results suggest that the marine environment not only serves as a transport pathway for industrially generated sulphate particles, but can also enhance the conversion of SO_2_ into SO_4_^2−^.

### 3.4 Influence of free-tropospheric air masses on sulphur-containing species

In this section, we present a classification of air masses based on their travel height, with the objective of examining if CMN is actually representative of the FT, and how the FT influence (FT_SRR_) affects the concentration of sulphur-containing species.

High-altitude mountaintop sites are often used in attempts to sample free-tropospheric air. By calculating FT_SRR_ using the PBL height as a threshold we found that on average 83% of the air mass sampled at CMN can be considered representative of the FT. This means that on average 17% of a specific air mass reaching CMN has been exposed the PBL while the remainder has resided in the FT. This number is slightly bigger compared to the 76% calculated for the Chacaltaya (CHC) station, a high-altitude observatory in the Bolivian Andes, in ref. 25. However, their study assumed a constant pseudo-PBL height, while we used the PBL height calculated by WRF do distinguish between PBL and FT. When applying a constant pseudo-PBL height of 750 m, our results align closely with theirs. It is important to note that when using a pseudo-PBL, the results can be sensitive to the chosen threshold. The optimal threshold may depend on site-specific factors, and different locations may require different values. Fig. A3 in the ESI† shows the distribution of FT_SRR_ for different thresholds (500 m, 750 m, and 1000 m), highlighting this variability.

**Fig. 9:**
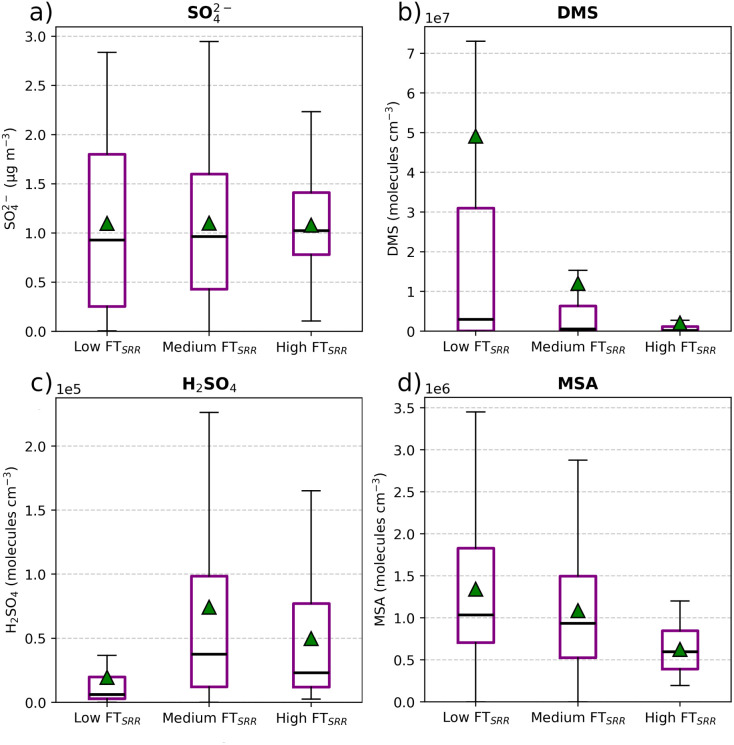
Boxplots of measured SO_4_^2−^ (a), and model DMS (b), H_2_SO_4_^2−^ (c), and MSA (d) in the low, medium and high FT_SRR_ categories. The central line in each box represents the median, the green triangle represents the mean, the box edges represent the IQR, and the whiskers extend to the smallest and largest values within 1.5 times the IQR. Any points outside the whiskers are considered outliers.

Different levels of FT_SRR_ can potentially impact the concentration of pollutants. Fig. 9 illustrates the concentrations of observed SO_4_^2−^ (a), modelled DMS, (b), modelled H_2_SO_4_ (c), and modelled MSA (d) across different air mass types. The classification is based on the FT_SRR_ categories introduced in the methods section.

The concentrations of SO_4_^2−^ exhibit no significant differences across the three categories. DMS and MSA concentrations show similar patterns across the three categories, with higher concentrations observed when the FT influence is smaller, indicating that DMS and MSA are primarily transported from the lower troposphere, with very little contribution from the FT. However, H_2_SO_4_ shows a different pattern, with lower concentrations when the PBL influence is higher. This is likely due to the lifetime of H_2_SO_4_ which strongly depends on the condensation sink (CS). In the PBL, aerosol concentrations are generally higher, providing more surface area for condensation. This elevated CS increases the removal of H_2_SO_4_, resulting in lower concentrations in air masses with a stronger influence from the PBL. All the patterns shown in Fig. 9 remain consistent even when using the other three thresholds used to calculate the FT_SRR_.

To contextualize these findings, we compare them with direct measurements of these compounds performed at CHC.^10^ As mentioned earlier, CHC's altitude (like that of CMN) makes it well-suited for studying the behaviour of atmospheric compounds in the FT with limited PBL influence. The main difference between our model results and the measurements collected at CHC is in the role of the FT. At CHC, DMS and MSA concentrations in the air masses arriving from the FT were notably higher compared to our findings at CMN. This discrepancy may be attributed to geographical and meteorological differences between the two stations. CHC is influenced by the Pacific Ocean, where stronger vertical convection could facilitate the transport of marine aerosols, including DMS and its oxidation products, into the FT. In contrast, CMN is primarily influenced by the Mediterranean Sea, which may exhibit less deep convection mixing, resulting in a reduced contribution of marine-derived compounds in the FT. Other important differences between the two locations, which could account for the differences in the FT influence, are altitude above sea level (CMN: 2165 m and CHC: 5421 m) and the distance from the sea (CMN: 50 km and CHC: 335 km).

## 4 Conclusions

In this study, we combined aerosol observational data with high-resolution Eulerian (WRF-CHIMERE) and Lagrangian (FLEXPART) modelling approaches to study the complex dynamics governing sulphate aerosol sources at the high-altitude Monte Cimone station during July 2017. Our analysis provides several key insights.

Marine areas were found to be a dominant source-region of sulphate. Air masses arriving at Monte Cimone frequently originate from or transit over the Mediterranean Sea. Additionally, extended exposure over the sea (quantified *via* ToS_500_) was found to be associated with enhanced hydroxyl radical (OH) concentrations, which in turn promote the oxidation of sulphur dioxide (SO_2_), eventually leading to the formation of sulphate aerosols.

The source-apportionment results showed a significant role of industrial emissions, despite the pronounced influence of marine areas. Sensitivity (zero-out) simulations reveal that industrial emissions remain a major contributor to sulphate at Monte Cimone. Industrial SO_2_, is significantly found even over the sea, where it is efficiently converted to sulphate under more favourable conditions (high OH and reduced dry deposition), highlighting the long-range impact of anthropogenic sources. This indicates that industrial emissions contribute significantly to both gaseous precursors and aerosol-phase components in marine air masses. Moreover, these results highlight the role of the marine environment as both an effective “producer” and “carrier” of secondary aerosols.

The analysis using the free-tropospheric influence metric (FT_SRR_) indicates that a large fraction of the sampled air masses are predominantly exposed to the free troposphere. Although overall sulphate concentrations are similar between different free-tropospheric influences, the behaviour of other sulphur-containing species suggests that vertical mixing processes between the boundary layer and the free troposphere significantly affect aerosol chemistry. Free-tropospheric air masses were found to have higher levels of sulphuric acid than air masses more exposed to the planetary boundary layer, while the opposite was found for DMS and MSA. This suggests that sulphate production might be governed by distinct chemical pathways depending on the degree of free-tropospheric influence: free tropospheric conditions favour formation from sulphuric acid, whereas boundary layer conditions could promote pathways associated with DMS oxidation.

This research calls for a holistic approach to air quality management that considers both marine and terrestrial emissions and the long-range meteorological transport of pollutants. Future studies should continue to explore the interactions between different emission sources, atmospheric chemistry, and meteorological factors to improve our understanding of aerosol dynamics in diverse environments. This work contributes to the broader understanding of aerosol dynamics in complex terrains by highlighting the importance of combined observational and modelling approaches.

A limitation of our study is that it focuses on a single month, and transport pathways and dynamics could vary in different seasons or under different meteorological conditions. Future studies should extend the analysis to multiple time periods to assess the robustness of our findings and capture potential seasonal variations in sulphate aerosol sources and transport dynamics. Also, further investigations should focus on the role of the different processes at different scales, particularly those at smaller scales affecting uptake, deposition and mixing within the atmospheric boundary layer, and assess to what extent they are appropriately reproduced by turbulence parameterizations available in the numerical models.

## Author contributions

G. C. designed the study. M. B. performed the FLEXPART simulations and all data analysis. B. V. prepared the WRF-CHIMERE model input data, and ran all of the WRF-CHIMERE simulations. A. C. provided extensive support in running the WRF-CHIMERE model and preparing the model data. A. C., D. Z., J. M., V. S., P. C., A. M., M. M., L. H., M. A., M. P., B. B., P. T., and F. B. participated in data collection and/or discussion of the analysis. M. B. and G. C. prepared the first version of the manuscript with contributions from all co-authors. All authors reviewed the manuscript and agreed on the final version of the paper.

## Conflicts of interest

The corresponding author has declared that none of the authors has any competing interests.

## Supplementary Material

EA-005-D5EA00035A-s001

## Data Availability

Sulphate observational data, FLEXPART output and sulphate output for all the simulations used here are all available at the following repository: https://doi.org/10.5281/zenodo.15055776. The source code of WRF-CHIMERE can be downloaded from https://www.lmd.polytechnique.fr/chimere/. The source code of WRF-FLEXPART can be downloaded from https://git.nilu.no/flexpart/flexpart-wrf. All other WRF-CHIMERE output data can be obtained upon request from the corresponding authors.
