# Supplementary material for: Across land, sea, and mountains: sulphate aerosol sources and transport dynamics over the northern Apennines
Source: Environ Sci Atmos. 2025 Jun 30;5(9):1023–34. doi: 10.1039/d5ea00035a (PMC12261285; doi:10.1039/d5ea00035a)
Supplement: EA-005-D5EA00035A-s001 [file EA-005-D5EA00035A-s001.pdf]

Supplementary Information (SI) for Environmental Science: Atmospheres.
This journal is © The Royal Society of Chemistry 2025

Table A1. GNFR sectors with their description.

GNFR	Code	Description
1	A_PublicPower	Emissions from plants producing electricity and/or heat for the public grid.
2	B_Industry	Emissions from combustion and processes in industry.
3	C_OtherStationaryComb	Emissions from small combustion sectors, e.g. commercial, institutional, residential and agricultural.
4	D_Fugitives	Fugitive emissions associated with production, refining, transport and storage of fuels.
5	E_Solvents	Emissions from the use of solvents.
6	F_RoadTransport	Emissions from road transport.
7	G_Shipping	Emissions from domestic navigation, i.e. navigation between two domestic ports. Fishing is included under I_OffRoad.
8	H_Aviation	Emissions from landing and take-off (LTO) both for domestic and international flights.
9	I_OffRoad	Emissions from machinery used in industry, households, agriculture as well as from railways and fishing vessels.
10	J_Waste	Emissions associated with waste handling. Waste incineration with energy recovery is included under A_PublicPower or B_Industry.
11	K_AgriLivestock	Emissions associated with animal husbandry and manure management.
12	L_AgriOther	All other agricultural emissions, e.g. from application of mineral or organic fertilizer, crops and field operations.
13	N_Natural	Emissions from natural sources, e.g. volcanoes, forest fires, etc.
14	O_AviCruise	Emissions from the cruise phase of both domestic and international flights.
15	P_IntShipping	Emissions from international navigation.

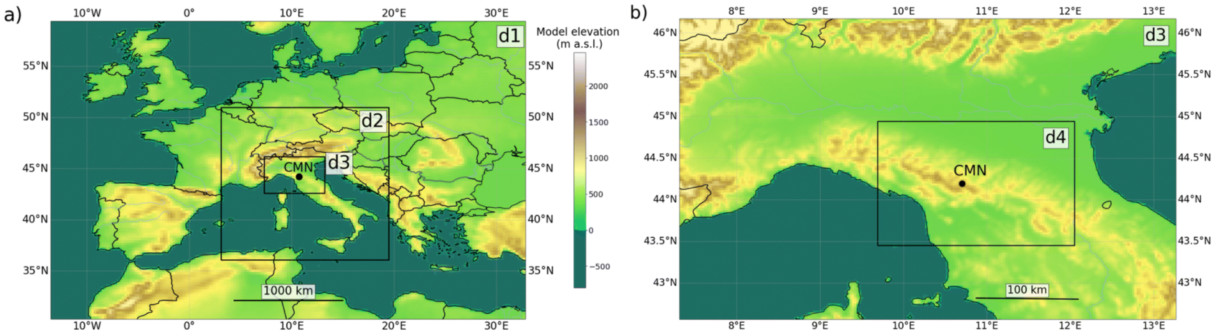

Figure A1. a) Position and topography (color shading) of the model domains d1 (27 km resolution), d2 (9 km resolution) and d3 (3 km resolution), (b) zoom-in of domains d3 and d4 (1 km res.). The dot indicates the location of the measurement station. From Vitali et al., 2024.

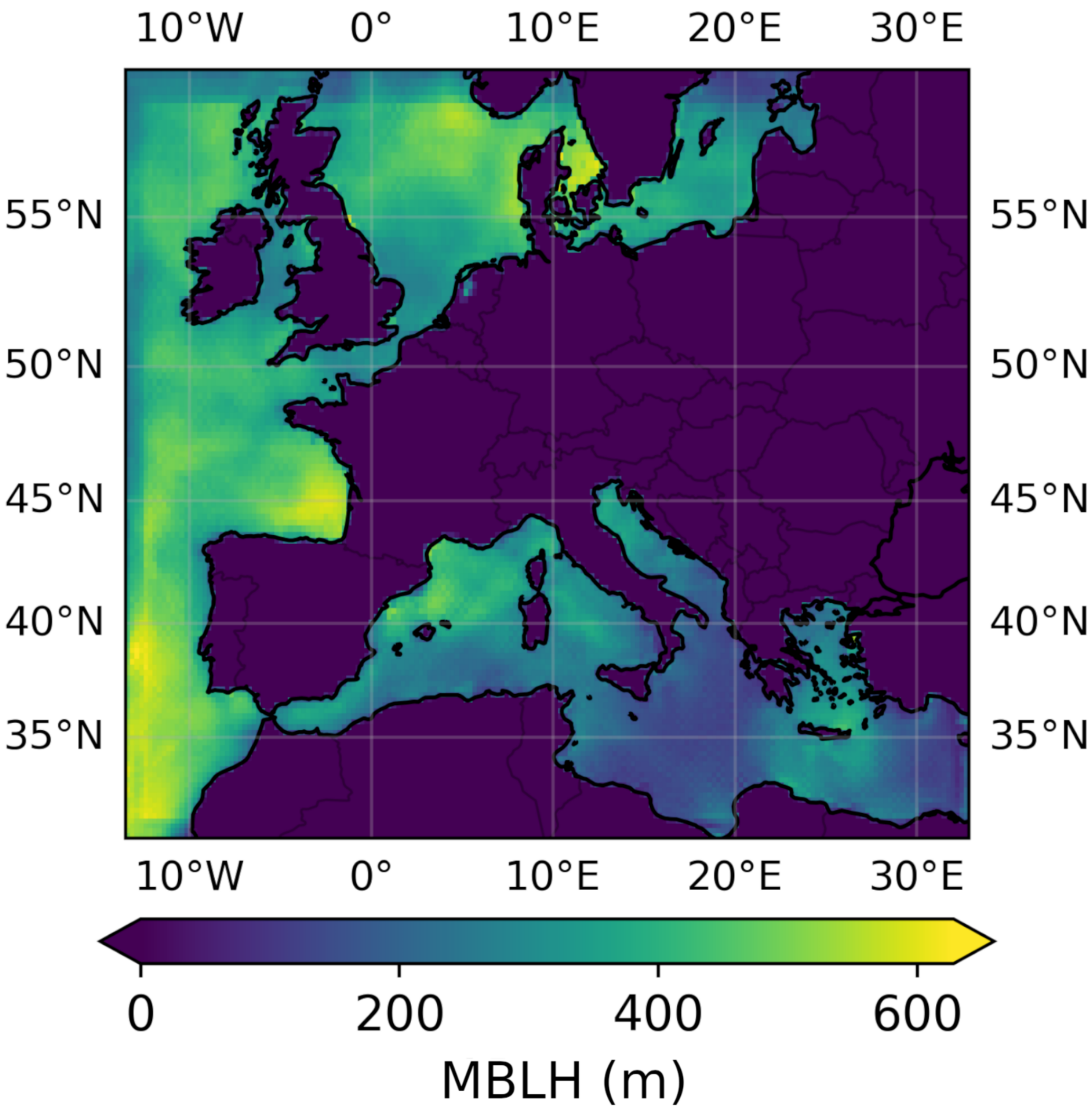

Figure A2. Average marine boundary layer height (MBLH) as calculated by WRF.

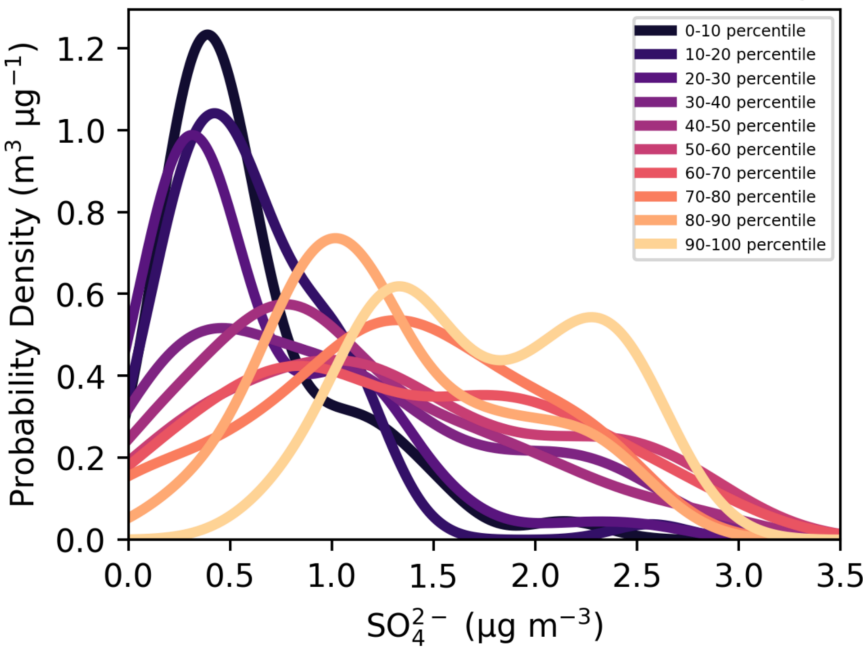

Figure A3. SO_4^{2-} distribution at CMN based on the number of hours air masses spent above the sea (below 500 meters altitude) during the 72 hours prior to reaching the site. Each categories represent a decile.

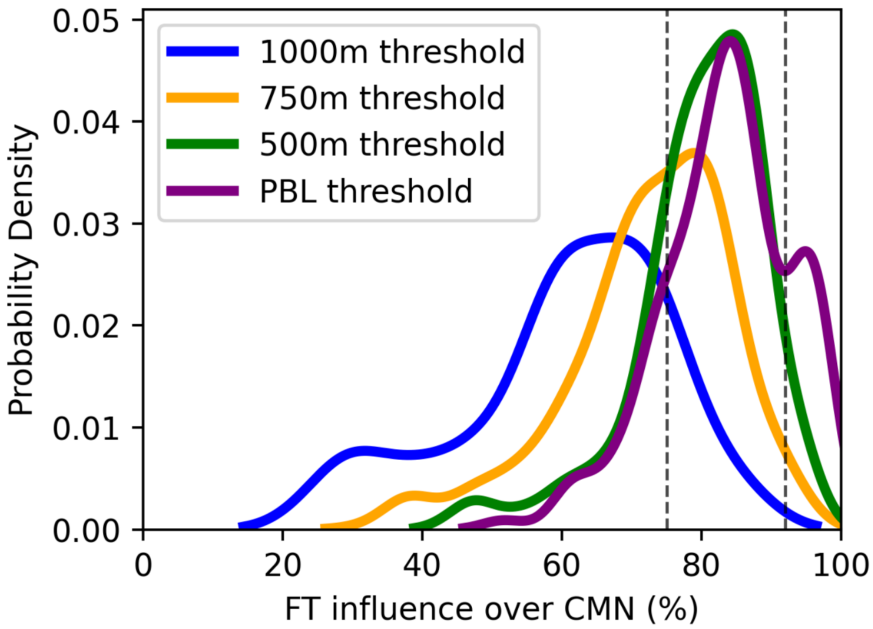

Figure A4. FT influence (FT_{SRR}) distribution calculated using different constant pseudo-PBL height as threshold (blue, orange and green line), and by using a changing threshold based on the PBL height calculated by WRF (purple line). The two vertical dashed line represent the division between the FT_{SRR} categories used in the analysis.

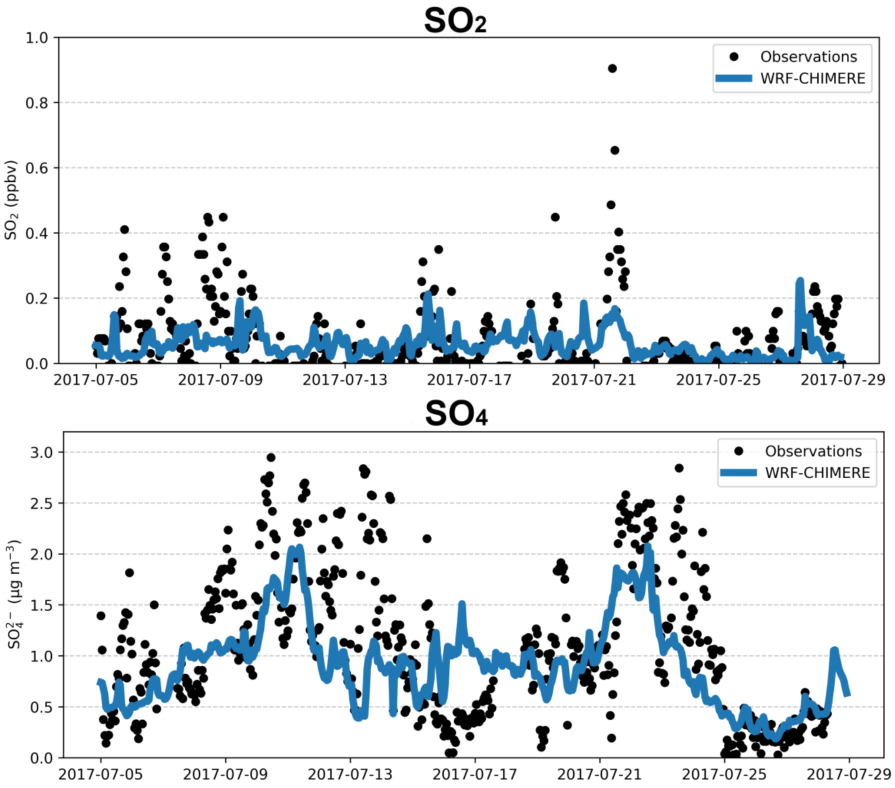

Figure A5. Hourly timeseries of the modelled (blue line) and observed (black dots) sulphur dioxide (top plot) and sulphate (bottom plot) concentration.

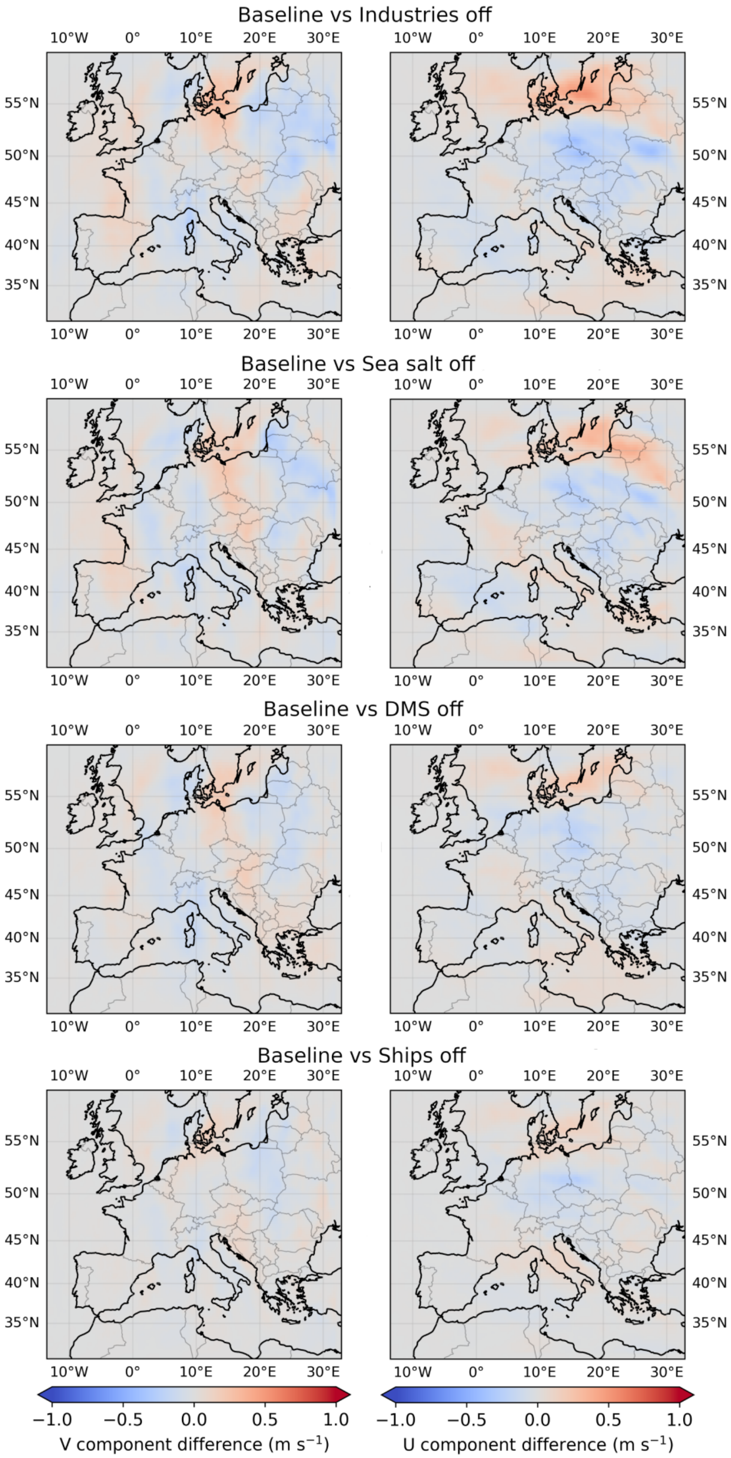

Figure A6. Average wind components difference between baseline simulation and the sensitivity simulations.
